# Mining of Textual Health Information from Reddit: Analysis of Chronic Diseases With Extracted Entities and Their Relations

**DOI:** 10.2196/12876

**Published:** 2019-06-13

**Authors:** Vasiliki Foufi, Tatsawan Timakum, Christophe Gaudet-Blavignac, Christian Lovis, Min Song

**Affiliations:** 1 Division of Medical Information Sciences University Hospitals of Geneva Geneva Switzerland; 2 Faculty of Medicine University of Geneva Geneva Switzerland; 3 Department of Library and Information Science Yonsei University Seoul Republic of Korea

**Keywords:** social media, chronic disease, data mining

## Abstract

**Background:**

Social media platforms constitute a rich data source for natural language processing tasks such as named entity recognition, relation extraction, and sentiment analysis. In particular, social media platforms about health provide a different insight into patient’s experiences with diseases and treatment than those found in the scientific literature.

**Objective:**

This paper aimed to report a study of entities related to chronic diseases and their relation in user-generated text posts. The major focus of our research is the study of biomedical entities found in health social media platforms and their relations and the way people suffering from chronic diseases express themselves.

**Methods:**

We collected a corpus of 17,624 text posts from disease-specific subreddits of the social news and discussion website Reddit. For entity and relation extraction from this corpus, we employed the PKDE4J tool developed by Song et al (2015). PKDE4J is a text mining system that integrates dictionary-based entity extraction and rule-based relation extraction in a highly flexible and extensible framework.

**Results:**

Using PKDE4J, we extracted 2 types of entities and relations: biomedical entities and relations and subject-predicate-object entity relations. In total, 82,138 entities and 30,341 relation pairs were extracted from the Reddit dataset. The most highly mentioned entities were those related to oncological disease (2884 occurrences of cancer) and asthma (2180 occurrences). The relation pair anatomy-disease was the most frequent (5550 occurrences), the highest frequent entities in this pair being cancer and lymph. The manual validation of the extracted entities showed a very good performance of the system at the entity extraction task (3682/5151, 71.48% extracted entities were correctly labeled).

**Conclusions:**

This study showed that people are eager to share their personal experience with chronic diseases on social media platforms despite possible privacy and security issues. The results reported in this paper are promising and demonstrate the need for more in-depth studies on the way patients with chronic diseases express themselves on social media platforms.

## Introduction

### Background

People are often concerned about their health status and a range of medical issues, especially when it comes to complex or chronic diseases that can take a long time to treat or monitor. Patients often desire easy access to information about diseases and symptoms to understand their condition and to facilitate self-management of diseases without total reliance upon interaction with a physician [1]. Patients with chronic diseases in particular use social media to seek and provide social, emotional, and practical support [2]. Therefore, social media information can influence patients’ decisions to manage their chronic condition [3].

Social media platforms may support patients in their search for medical products or provide suggestions to promote healthy behavior and can improve health education as they allow people to write about their experiences with diseases, drugs, symptoms, and treatments. In recent years, social media platforms have grown quickly, with the public, patients, and health professionals sharing their experiences, looking for information, and interacting with others.

Currently, more than 74% of internet users connect to social media, and 42% of the internet users take advantage of social media for health information. Moreover, 32% of social media users in the United States share about their health care experiences and family’s struggle stories and 29% search for health information via social media platforms to observe other patients’ experiences with their diseases [3]. Furthermore, 51% of those who live with a chronic disease have used the internet for information about health topics such as details of a specific disease, medical procedures, drugs, medical devices, or health insurances [4].

With its growing number of users, social media has become a powerful tool that can promote information sharing about health care, provide feedback from users, and foster support systems [5]. In addition, the existence of social media platforms enables researchers to learn and discover the health experiences and feeling of patients and potentially discover new knowledge in health science. For example, user conversation content from health-related online forums, such as blogs, Twitter, and Facebook, has already been analyzed to find the clusters of breast cancer symptom [6], examine smoking [7], and understand the user discourse and describe social media interactions about obesity prevention [8]. In particular, Reddit has been used as a data source for similar studies [9-12].

The interactions between individuals on social media and the information they share constitute an important new source of data that can be used, on one hand, to understand the impact of drugs, diseases, and medical treatments on patients outside controlled clinical settings and, on the other hand, to comprehend health-related behavior.

Discovering public knowledge in social media text constitutes a challenge for researchers and health care providers. To achieve this goal, various text mining approaches, such as topic modeling, information extraction, and visualization, exist.

### Biomedical Entity and Relation Extraction

In the era of biomedical text mining, bioentities and their relations have arisen as a challenge to discover new knowledge. To mine the huge amounts of unstructured data, automatic information extraction tools have been conceived and developed based on several approaches. There are multiple systems developed for the identification and analysis of relations between diseases, drugs, and genes, such as Extraction of Drugs, Genes and Relations, a natural language system that extracts information about drugs and genes relevant to cancer from the biomedical literature [13]. Extraction of drug-disease treatment pairs from the published literature was also carried out [14,15]. To extract health social media information, adverse drug reactions and drug indications from a Spanish health forum were examined [16] using MeaningCloud [17], a multilingual text analysis engine based on a distant-supervision method to detect relations between drugs and side effects and used them to classify the relation instances.

PKDE4J2.0 is a system that extracts bioentities and their relations with the aim to discover biomedical scientific knowledge. It is based on a dictionary to automatically tag bioentities according to their types and a set of predefined rules used for relation extraction. PKDE4J2.0 can be applied for knowledge search, knowledge network construction, and knowledge inference [18]. PKDE4J1.1 was used to investigate drug-disease interactions in article abstracts from PubMed Central for making drug-symptom-disease triples [19]. This tool was also applied in biomedical literature to extract biomedical verbs to present a relation type between 2 entities [20] and on full-text papers to extract biological entities from diseases and genes and construct a knowledge network [21].

### Health Information Extraction From Social Media Platforms

A large number of patients, caregivers, and health professionals use social media platforms to discuss mental health issues. They also constitute an important data source for researchers. Machine learning and statistical methods were used to discriminate online messages between depression and control communities using mood, psycholinguistic processes, and content topics extracted from the posts generated by members of these communities [22]. Users are interested in searching for treatment-related information, communicating with physicians to share their feelings about treatment effectiveness and side effects, discussing questions in health communities, and gaining knowledge about their conditions [23]. User-generated content from these platforms contains valuable information [24]. Their posts reflect what users think and feel about their medical experiences and often attract the attention of other patients, caregivers, and doctors.

Lu et al [25] mined data from online health communities and used text clustering integrating medical domain–specific knowledge to investigate patient needs and interests. Their results show that compared with existing methods, the addition of medical domain–specific features into their feature sets achieved significantly better clustering than was achieved without the addition of those features. Moreover, there were significant differences in hot topics on different kinds of disease discussion platforms. Health-related posts on social media were analyzed to investigate the polarity of opinions online, performing sentiment analysis [26]. Medical terms, including those related to conditions, symptoms, treatments, effectiveness, and side effects, were extracted to generate a virtual document addressing each question raised by members of the community. Then latent Dirichlet allocation (LDA) was modified by adding a weighting scheme known as conditional LDA to cluster virtual documents with similar distributions of medical terms into a conditional topic (C-topic). Finally, the clustered C-topics were analyzed according to sentiment polarities and physiological and psychological sentiments. Identification of topics of patients' discussions on (1) Facebook about breast cancer and (2) cancerdusein.org was performed [27]. These topics were assigned to functional and symptomatic dimensions by applying LDA topic modeling and identified relations between the topics and the questionnaires.

Among others, Denecke [1] reported that “user-generated content on the web has become a new source of useful information to be added to the conventional methods of collecting clinical data.”

In terms of biomedical information extraction, previous studies relied on formal research and individual case studies to identify biomedical information. These approaches include observations of changes in patients [28], meta-analysis of data from relevant databases [29], and surveys of cancer patients [30]. However, the scientific literature is generally limited to subscribers, and electronic medical records are not publicly available for reasons of patient privacy [31]. Moreover, these sources do not provide a complete understanding of how patients suffering from a chronic disease feel and how they express these feelings.

Using data from conversations between patients on social media platforms provides valuable information for researchers, physicians, and health care providers. This data source is different from, and complementary to, that obtained from conventional experimental methods.

### Research Objectives

Therefore, a social media platform (Reddit) was chosen as the data source for this research that aimed to answer the following questions:

1. Which biomedical entities are prominent in the health social media platforms?
2. What types of entities are related in the corpus?
3. How do people express themselves about chronic diseases on social media platforms?

## Methods

### Data Collection

The data used for this research were extracted from disease-specific subreddits of the social news and discussion website Reddit [32]. Forums such as Reddit tend to have sharp contrast when compared with similar offline groups; for instance, people are likely to discuss problems that they do not feel comfortable to discuss face to face [33]. As of 2013, Reddit’s official statistics included 56 billion page views, 731 million unique visitors, 40,855,032 posts, and 404,603,286 comments [34]. In particular, the subreddit about cancer numbers 22,429 subscribers and 75 posts per day [35]. These numbers demonstrate the external validity of Reddit. Another reason for having chosen Reddit as a data source is that the language of text posts is more structured than in other social media platforms such as Twitter.

Reddit’s core functionality is the sharing of text-based posts with others who may or may not be members of the site. The subforum function allows the creation of designated spaces for users to congregate and interact with each other over a shared interest. Those subforums are called *subreddits*. A finite set of 19 subreddits related to chronic diseases was empirically selected for analysis. The choice of the specific subreddits was based on medical expertise and on the impact of these diseases on the quality of everyday life of patients.

As the main goal was the detection of relations between entities and of the way people suffering from chronic diseases express themselves in social media and not the study of characteristics of specific chronic diseases, the posts from the 19 subreddits were merged in a single dataset.

All of these subreddits host public content. In this research, no populational study has been performed. The study focuses on the expression of feelings and not on the identity of people sharing their experiences. From each post, only the title of the post and the body or textual content was extracted without additional information related to their authors.

The study was submitted to the Swiss Ethical Committee who concluded to a decision of nonconsideration provided that the collected data are not identifiable.

### Lexicosemantic Resources

Lexicosemantic resources were constructed and incorporated into the tool. These resources included a list of stop words and biomedical dictionaries of diseases, drugs, anatomy, procedures, symptoms, side effects, and findings created from clinical health care terminologies such as the Systematized Nomenclature of Human and Veterinary Medicine - Clinical Terms [36], the National Library of Medicine's controlled vocabulary thesaurus [37], the Gene Ontology knowledgebase [38], the Kyoto Encyclopedia of Genes and Genomes database [39], and the DrugBank database [40]. Semantic relations properties were attributed to 4558 biomedical verbs extracted from the Unified Medical Language System [41].

The dictionaries were enriched with lemmas extracted from the corpus; for instance, *chemo*, *AML* (acute myeloid leukemia), *take care, support*, and *fight*.

### Description of the Tool

In this research, the PKDE4J version 2.0 tool [42] was used. This text mining system consists of 2 modules: entity extraction and relation extraction.

#### Entity Extraction Module

This module integrates dictionary-based entity extraction and rule-based relation extraction into a highly flexible and extensible framework. The Stanford CoreNLP pipeline [43] was modified to make it suitable for advanced dictionary-based entity extraction. The entity extraction module consists of 4 major submodules: preprocessing, dictionary loading, entity annotation, and postprocessing. PKDE4J can analyze entities and relations from both structured and unstructured text.

#### Relation Extraction Module

**Figure 1:**
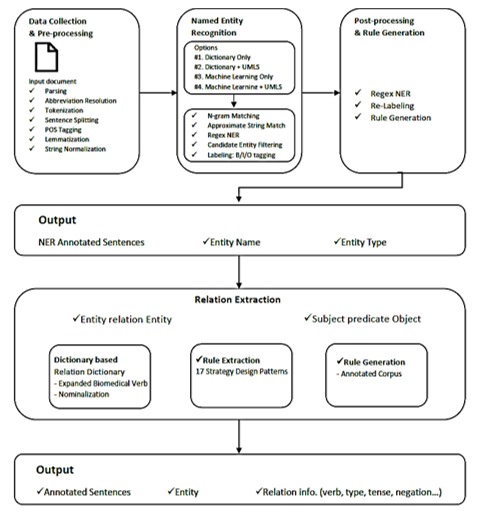
The workflow of the PKDE4J text mining system.

The relation extraction workflow identifies directed qualified relations starting from sentences from which 2 or more entities have been extracted by the entity extraction module. The relation extraction module takes a list of verbs and nominalization terms that are employed to identify relations of interest. After extracting entities from a sentence, further relation extraction algorithms are executed to construct rules for the extraction of relations of entities. A set of 20 dependency parsing–based rules is at the core of the relation extraction module and provides an ontologically enriched structure for sentences by annotating edges with dependency types. To extract relations, the system identifies a verb, which may be located between entities and contains relational characteristics, then, it checks the bioverb list to determine the relation between the entities (Figure 1) [44].

### Visualization

The Gephi platform [45] was used to visualize the network of chronic diseases in the corpus. To build a graph, the *k-* shortest paths routing algorithm was applied. The graph visualization tool was then used to map the chronic disease entities. A PageRank of terms was computed to rank the important entities in the network; therefore, entities ranked highly by PageRank have the highest impact.

### Validation of Entity Extraction

To evaluate the performance of the tool on entity extraction, 1000 posts randomly selected from the entire corpus were manually validated. The entities were evaluated as correct or incorrect based on the following specific guidelines.

#### Findings and Symptoms

This category refers to a phenomenon that is experienced by a person or described by a clinician and cannot be considered as a disease in the context, for example, “This news makes me feel anxiety.”

#### Disease Names

This category refers to an abnormal condition of a human, animal, or plant that causes discomfort or dysfunction [46]. As also mentioned in the previous category, the context helps to distinguish between a disease and a symptom or finding. For example, in the sentence “After trying which dosage is good, my insomnia is thankfully gone again,” *insomnia* refers to a disease, whereas in the sentence “I have had symptoms of insomnia within the last months,” *insomnia* describes a symptom/finding.

#### Side Effects

This category includes a symptom/finding or a disease that is caused by a treatment in the context. For example:

Since beginning treatment have woken with bouts of nausea...

#### Procedure

Procedure refers to any intervention carried on someone related to physical mental or social health. For example:

...treatment which would include surgery and radiation/chemotherapy according to his oncologist

## Results

### Data Collection

A dataset of 17,624 text posts was semiautomatically collected using crawlers accessing public streams. Table 1 shows the subreddits used for this research, the number of posts per subreddit, and the proportion of corpus representation of each subreddit:

**Table 1:** Sources used for the data collection.

Subreddit name	Number of posts	Proportion of posts from each subreddit in the corpus
r/cancer	5210	26.9
r/MultipleSclerosis	1902	9.8
r/rheumatoid	1783	9.2
r/CrohnsDisease	1722	8.9
r/Asthma	1600	8.3
r/testicularcancer	1384	7.1
r/Parkinsons	1042	5.4
r/Hashimotos	1022	5.3
r/Alzheimers	927	4.8
r/breastcancer	794	4.1
r/braincancer	623	3.2
r/pancreaticcancer	397	2.1
r/lymphoma	387	2.0
r/leukemia	223	1.2
r/kidney	107	0.6
r/multiplemyeloma	104	0.5
r/thyroidcancer	63	0.3
r/lungcancer	41	0.2
r/skincancer	15	0.1
Total	19,346	100

After sorting the data corpus, duplicate posts and those with no relevant meaning, such as advertising posts and posts containing only a hyperlink, were removed. The final corpus comprises 17,580 posts (2,137,115 tokens).

### Biomedical Entity and Relation Extraction

The PKDE4J system performed named entity extraction and 2 types of relation extraction: relations between biomedical entities and between subjects, predicates, and objects on the sentence level. The system’s output is a corpus annotated with entities and information about their relation.

The entities are either simple terms or complex structures referring to diseases, anatomy, procedures, findings, symptoms, side effects, or drugs. In total, PKDE4J extracted 82,138 entities from the Reddit dataset, as shown in Table 2. The entity names and entity types were allocated to the 7 categories of the biomedical dictionaries. The 10 most frequent entity names followed by the number of occurrences in the corpus are displayed in Table 3. It should be noted that the terms are given in the text in the form found in the corpus. Therefore, abbreviated terms have not been expanded.

As displayed in the table, 29,669 disease entities were extracted representing 1341 unique diseases; 19,956 anatomy entities, of which 369 are distinct anatomical terms; 11,549 procedures of 296 different types; 6256 symptoms entities describing 65 symptoms; 5351 entities representing side effects of 321 different types; and 35 different drug names (616 in total). The most highly represented diseases are oncological (*cancer*, *breast cancer*, *tumor*, *leukemia*, and *lymphoma*) or relate to asthma. The anatomy category contains a range of anatomical terms. Specifically, *blood* is the most frequent term. Other widely used anatomical terms are *back*, *brain*, *hand*, *hair*, *breast*, *chest*, *heart*, and *neck*. The procedures category comprises terms referring to chemical treatments (*chemo*), surgery, laboratory test (*blood test*), social interventions (*advice* and *listening*), and others.

The most frequent symptom mentioned in the corpus is *pain* (472 occurrences). *Fatigue*, *inflammation*, *nausea*, and *cough* are some of the symptoms commonly reported by patients or relatives in the dataset. In the side-effect category, the most frequent entities are *anxiety*, *stress*, *swelling*, *crying*, and *fear* followed by *disability* and *worry*. The most commonly reported drug is *prednisone* followed by *morphine*, *salbutamol*, and *tramadol*.

### Validation of Entity Extraction

Among the 5151 extracted entities, 3682 were correctly labeled by the system, whereas 1469 were attributed with incorrect labels. The performance of the system was 71.48%.

Next, an error analysis was performed on the incorrectly labeled entities. Errors were classified into 3 categories:

1. Lexical errors (488/1469, 33.21%): the term *breast* is an anatomical term, but in the post, the compound term *breast cancer* appears. However, the system failed to extract the entire entity.
2. Dictionary errors (550/1469, 37.44%), for example, *air* and *aspergillus* were falsely listed as an anatomical term and as a drug name, respectively.
3. Ambiguous concepts (431/1469, 29.33%): the term *bleeding* could be either a disease name or a symptom.

**Table 2:** Entity extraction results.

Entity types	Diseases	Anatomy	Procedures	Findings	Symptoms	Side effects	Drugs
Extracted entities, n	29,669	19,956	11,549	8741	6256	5351	616
Entity names, n	1341	369	296	483	65	321	35

**Table 3:** Ten most frequent entities by type.

Diseases	Anatomy	Procedures	Findings	Symptoms	Side effects	Drugs
Cancer (2884)	Blood (1542)	Chemo (1914)	Related (521)	Pain (2648)	Anxiety (683)	Prednisone (417)
Asthma (2180)	Back (1034)	Treatment (1909)	Lump (359)	Fatigue (639)	Stress (373)	Morphine (33)
All (2163)	Brain (962)	Surgery (1909)	Suffering (333)	Inflammation (472)	Swelling (348)	Salbutamol (33)
Breast cancer (804)	Hand (656)	Advice (774)	Confused (305)	Scared (273)	Crying (245)	Tramadol (26)
Can (745)	Head (627)	Radiation (627)	Problem (304)	Nausea (244)	Mass (220)	MRSA^a^ (14)
Tumor (631)	Hair (549)	Biopsy (366)	Attack (277)	Hurt (205)	Fear (215)	Aspirin (11)
Disease (563)	Breast (535)	Chemotherapy (338)	Energy (270)	Sore (202)	Disability (164)	Omeprazole (9)
TSH^b^ (506)	Chest (511)	Blood test (268)	Terrified (266)	Numb (195)	Worry (142)	Seretide (6)
Depression (414)	Heart (503)	Infusion (199)	Tired (251)	Cutting (104)	Fall (129)	Citrus (5)
Lymphoma (348)	Neck (459)	Listening (158)	Follow up (249)	Tingling (101)	Discomfort (120)	Echinacea (5)

^a^MRSA: methicillin-resistant *Staphylococcus aureus*.

^b^TSH: thyroid stimulating hormone.

### Relation Extraction

The system extracted 2 entities (entity 1 and entity 2) found in the same sentence and linked with a relation and then it attributed the type of entities. For instance, entity 1, *Borderline* (disease) co-occurs with *High blood pressure* (symptom) in the sentence. In total, 30,341 relation pairs were extracted, as shown in Table 4.

Of the 30,341 relation pairs, the most frequent entity relation pairs and their number of co-occurrences are shown in Table 5.

The relations between anatomy and disease entity types are the most frequent (5550 pairs). The pair disease-disease co-occurs 4668 times, and the pair anatomy-anatomy appears 3595 times.

Table 6 contains the 5 most frequent entities per relation pair.

**Table 4:** Example of entity relation extraction.

Analysis result	Entity 1	Entity 1 type	Entity 2	Entity 2 type	Sentence from post
Output 1	Borderline	disease	High blood pressure	symptom	Prior to that, I was fat mid forties male borderline high HDL, high blood pressure but ZERO issues with thyroid or immune issues.
Output 2	Optic neuritis	disease	Multiple sclerosis	symptom	She said that I have something called optic neuritis and that about half the time people get it and they don’t know why but the other half its because someone has multiple sclerosis.
Output 3	Syndrome	disease	Nerve	anatomy	I had bilateral optic neuritis significantly worse in my left eye in Late August September and I was also simultaneously diagnosed with Browns Syndrome which they’re not 100% convinced on as it may have been misdiagnosed 6th nerve palsy.

**Table 5:** Most frequent entities per relation pair.

Relation pair	Co-occurrences, n
anatomy-anatomy	3595
anatomy-disease, disease-anatomy	5550
anatomy-procedure, procedure-anatomy	1730
anatomy-symptom, symptom-anatomy	1227
anatomy-side effect, side effect-anatomy	1081
disease-disease	4668
disease-procedure, procedure-disease	2540
disease-finding, finding-disease	2128
disease-side effect, side effect-disease	1502
disease-symptom, symptom-disease	1080
finding-finding	303
finding-anatomy, anatomy-finding	1362
procedure-procedure	1023
procedure-finding, finding-procedure	430
side effect-side effect	256

**Table 6:** The 5 most frequent entities per relation pair.

Rank	Pair of entity 1 and entity 2					
	Anatomy|Anatomy	Disease|Disease	Disease|Side effect	Disease|Anatomy	Disease|Procedure	Disease|Finding
1	Back|Hair	Cancer|ALL	Depression|Anxiety	Cancer|Lymph	Cancer|Surgery	Asthma|Attack
2	Neck|Lymph	Asthma|Allergy	Asthma|Anxiety	Tumor|Blood	Tumor|Surgery	Cancer|Suffering
3	Brain|Lungs	Tumor|Seminoma	Cancer|Fear	Asthma|Lungs	Breast cancer|Treatment	ALL|Follow up
4	Lungs|Lymph	ALL|Asthma	ALL|Swelling	ALL|Blood	Asthma|Advice	Depression|Suffering
5	Head|Hair	Depression|Fatigue	Aches|Anxiety	TSH|Blood	ALL|Treatment	Exercise|Muscle tension

The most frequent entities in the pair disease-anatomy is *Cancer|Lymph*, in the pair disease-disease is *Cancer|ALL*, and in the pair anatomy-anatomy is *Back|Hair*.

**Figure 2:**
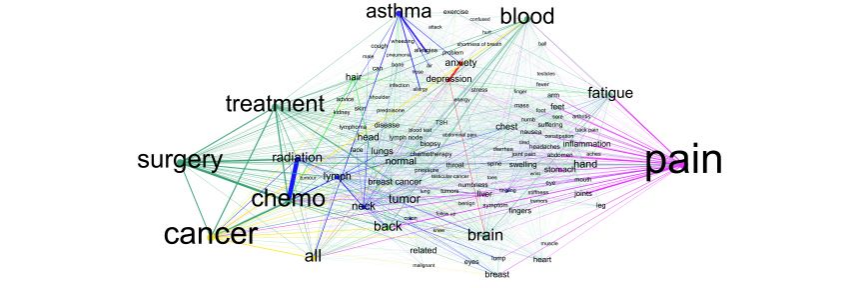
Biomedical entity network.

To summarize, once the most frequent entities were extracted, the results were processed according to the shortest path between each entity pair to produce the graph shown in Figure 2. Among 2561 nodes and 13,405 edges, this entity network shows that *pain* highly co-occurs with other entities in the network (biggest node, weighted at 0.022461), followed by *cancer* (PageRank score at 0.018057) and *surgery* (PageRank score at 0.015443).

The node *pain* has connections with other nodes, including *fatigue*, *inflammation*, *stomach*, *joints*, *cancer*, and *chemo*. The node *cancer* is strongly linked to *chemo*, *surgery*, *treatment*, *ALL (acute lymphoblastic leukemia)*, *anxiety*, and *blood*. The nodes of *surgery*, *chemo*, and *treatment* are linked to diseases and body parts. Finally, the entity nodes relating to mental health, such as *anxiety* and *depression*, also appear in the network and associate with other bioentity types. Table 7 shows the most frequent entities and the corresponding PageRank scores:

**Table 7:** The most frequent entities and the corresponding PageRank scores.

Label	PageRank score
Pain	0.022461
Cancer	0.018057
Surgery	0.015443
Chemo	0.014954
Treatment	0.014275
Blood	0.013841
Asthma	0.012554
All	0.010931
Brain	0.010311
Fatigue	0.009626
Back	0.008574
Radiation	0.008317
Tumor	0.007814
Neck	0.006968
Hand	0.006618
Lymph	0.006442
Normal	0.006176
Anxiety	0.006108
Head	0.005933
Hair	0.005762

### Subject-Predicate-Object Entity Relation Extraction

The system extracted 69,263 subject or object entities. The top 10 entities are shown in Table 8. In total, 41,068 relations were extracted and the results were classified into 2 types of subjects: subject pronoun (*I, you, he, she, it, we*, and *they*) and subject noun (*treatment*). The relation pairs are divided into 19,645 pairs of subject pronoun-object entities and 21,423 pairs of subject noun-object entities.

Table 9 shows 2 examples of the subject-predicate-object relation extraction: the subject (for example *I*, *he*, *anyone*, *it*, and *asthma*), the predicate (verbs such as *have*, *get*, and *increase*), the object (terms such as *eczema*, *allergies*, *childhood asthma*, *my cough*, and *allergy shots*), and the sentence of the corresponding post.

#### Subject Pronoun-Predicate-Object

The subject pronoun-predicate-object relation extraction demonstrates that the most frequent subject pronoun is *I* (11,691 times, including *I’ve*, *I’m*, and *I’d*). Some examples are shown in Table 10.

#### Subject Noun-Predicate-Object

Table 11 shows some examples of subject noun-predicate-object relation extraction. Among the 21,423 relation pairs, the most frequent subject nouns are diseases such as *asthma* (272 occurrences), including phrases such as *asthma anxiety*, *asthma attacks*, *my asthma*, *my asthma and allergies*, *my asthma flare*, and *cancer* (226 occurrences).

**Table 8:** The top 10 occurrences of subjects and objects.

Entity	Count, n
I	10,314
It	2832
Pain	2341
She	1501
He	1323
Cancer	1060
Asthma	1015
They	900
Me	719
You	597

**Table 9:** Examples of subject-predicate-object relation extraction results.

Analysis result	Subject entity	Predicate	Object entity	Sentence
Output 1	I	Had	the skin allergy test	I had the skin allergy test done and it came back positive for almost every kind of pollen and mold, etc.
Output 2	my blue inhaler	Increases	my asthma	I noticed consistently my blue inhaler increases my asthma about 30% after using it and I believe was the cause of a recent very bad asthma attack.

**Table 10:** Example of subject pronoun-predicate-object relation extraction.

Subject	Predicate	Object	Sentence
I	take	the typical seretide	I take the typical seretide morning and night and ventolin when l need it.
I	have	a deep and painful cough	I have a deep and painful cough that's been leaving me with back, chest, and side pains.
You	ever take	allergy shots	Hey, to you asthmatics who have allergy induced asthma, did you ever take allergy shots.
He	was given	Prednisone	He was given prednisone for that as well.
She	has	Asthma	My sister, who lives with me, started complaining to me about it, saying that she doesn’t want me doing that when her daughter is home because she has asthma and it smokes up the house.

**Table 11:** Example of subject noun-predicate-object relation extraction.

Subject	Predicate	Object	Sentence
Asthma	is becoming way more than just	a physical issue	Asthma is becoming way more than just a physical issue, it's taking a toll on my mental health.
Cancer	had spread to	her bones	The doctor told her that cancer had spread to her bones and that she'll have to have injections for it?
Fever	is indeed mentioned as	a side effect	Ive also been using Modulair Montelukast Sodium, and fever is indeed mentioned as a side effect on my leaflet.
Hives	are from	allergies	They’re trying to tell me they are panic attacks but as far as I know hives are from allergies and they sometimes happen during my asthma attacks.
The depression	is occurring simultaneously with	the increased asthma symptoms	I noticed the depression is occurring simultaneously with the increased asthma symptoms and was wondering if there is a correlation and if anyone else has experienced this.
My milk allergy	was causing	my asthma	My milk allergy was causing my asthma.
My second course of prednisone	has been great for stopping	the wheezing	And I'm on my second course of prednisone which has been great for stopping the wheezing - even the rescue inhaler didn't help before.
The doctor	ruled out	pneumonia	Anyway, the doctor ruled out pneumonia and said I had caught a cold on the plane and it had triggered an asthma exacerbation.

**Figure 3:**
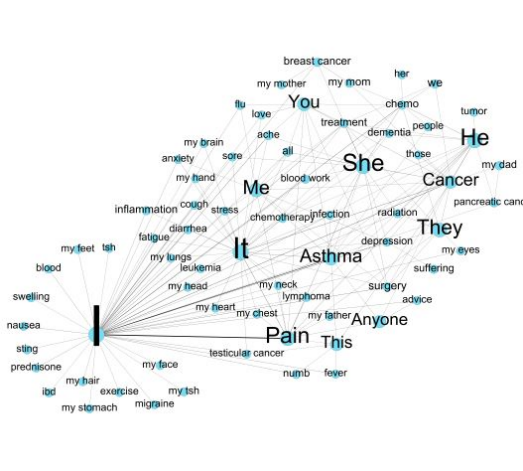
Subject and object entity network.

Figure 3 demonstrates the most used subject and object entities. The results show that the most frequent subject is the pronoun *I* (PageRank score: 0.100619). The pronoun *It*, *She*, *He*, *They*, and *You* are also frequently used. Diseases, body parts, treatments, and symptoms are widely used as the subjects and/or objects as well as the possessive pronoun *my* (*my mother*, *my eyes*, and *my dad*). Table 12 presents the most frequent subject and object entities and the corresponding PageRank scores.

### Social Media Language

Expressions that constitute specific terms developed on social media, such as *pm* (private message), *FWIW* (For What it's Worth) were identified in the corpus. “A common feature of microblog texts is the use of symbols in posts, such as the love-heart dingbat symbol” [47]. Emoticons such as 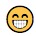, and text-based emoticons such as *LOL* (laughing out loud), :), :-(, =), and :( are also frequent.

In addition, the corpus contains informal phrases such as “Rooting for you!” and “I’m still chugging along”; adjectives such as *loopy*, *drippy*, *dicey*, and *zonked*; and verbs such as *puke* that substitute for their equivalents in standard language.

Entities found in the Reddit corpus present numerous morphosyntactic variants. For example, the term *chemotherapy* was rarely found, but the short form *chemo* was frequently used. The disease name *Hodgkin’s Lymphoma* is as *Hodgkin Lymphoma*, *Hodgkins Lymphoma*, *Hodgkin disease*, and *HL*. Similarly, the entity name *Mixed Cellularity Classical Hodgkin Lymphoma* is found as *Mixed Cellularity Hodgkin Lymphoma*, *Mixed Cellularity Hodgkins Lymphoma*, and *MCCHL.* Moreover, there are many abbreviated forms of entity names, such as *ALL*, *AML*, *BRCA2* (breast cancer type 2), *CLL* (chronic lymphocytic leukemia), *CML* (chronic myeloid leukemia), *COPD* (chronic obstructive pulmonary disease), *DCIS* (ductal carcinoma in situ), and *GERD* (gastroesophageal reflux disease). When these forms were included in the disease dictionary, the system managed to detect them. Some examples are presented in Table 13.

**Table 12:** The most frequent entities and the corresponding PageRank scores.

Label	PageRank score
I	0.100619
It	0.027234
Pain	0.020651
She	0.014206
He	0.012875
Cancer	0.00939
Asthma	0.009324
They	0.008927
Me	0.006934
You	0.005852

**Table 13:** Examples of disease entities in their abbreviated form.

Abbreviated form of entity name	Full entity name	Example from the corpus
ALL	Acute lymphoblastic leukemia	My boyfriend was diagnosed with ALL 2 years ago and stayed in remission after a few rounds of chemo.
AML	Acute myeloid leukemia	Diagnosed with AML this past Sept.
BRCA2	Breast cancer type 2	Her sister, my aunt, was diagnosed with breast cancer at 27 and was dead by 33 she tested positive for BRCA2 as well.
CLL	Chronic lymphocytic leukemia	My CLL is more of SLL, which is the same thing but presented in my lymph nodes.
CML	Chronic myeloid leukemia	25 years old, diagnosed with CML when I was 15.
COPD	Chronic obstructive pulmonary disease	Hes been smoking for over 40 years, has COPD and isnt in the greatest health generally overweight, inactive, etc.
DCIS	Ductal carcinoma in situ	We found out last week she has both DCIS and Invasive DCIS.
GERD	Gastroesophageal reflux disease	Sleep apnea can also worsen GERD, and GERD is known to worsen asthma.

## Discussion

### Principal Findings

In this paper, we collected user-generated chronic disease–related data from Reddit and extracted information pertinent to biomedical entities and their relations to examine the characteristics of the language used by users in this social media platform. Initially, the corpus was created by semiautomatically extracting posts from specific subforums of Reddit. Next, lexicosemantic resources from various sources were created. To perform the information extraction tasks—entity extraction and relation extraction—the PKDE4J text mining system was used. The system extracted 82,138 biomedical entities and 30,341 relations. These results indicate that the corpus contains a large amount of information.

### Performance of the Tool

As described in the Results section, the system achieved a high performance in the named entity extraction task and the attribution of entity types (3682/5151, 71.48% extracted entities were correctly labeled). As already mentioned, the language used in Reddit is structured enough, with a satisfactory number of full sentences so the system managed to extract entities and their relations. The error analysis showed that the system failed to detect a number of entities or falsely attributed the entity type, because of lexical errors, to dictionaries’ errors or to ambiguous concepts.

### Entity Extraction

Entities prominent in the corpus refer to diseases, anatomical terms, procedures, findings, and symptoms. While interpreting the entities extracted from the corpus, it must be taken into account that the corpus was constructed by selecting subreddits created to share information about specific diseases. Therefore, it is expected that entities related to these diseases are the most likely to be represented. For instance, parts of the body affected by specific cancers, such as *breast* or *blood*, occur very frequently.

The most frequent disease entities in this corpus are oncologic diseases such as *cancer*, *ALL*, and *breast cancer*. Frequently mentioned nononcologic diseases are *asthma*, *depression*, and the generic entity *disease*. The entity *thyroid stimulating hormone* (TSH) is frequently mentioned, but it should be further classified in findings.

The most frequent anatomy entity is *blood*. This is explained primarily because of the numerous posts speaking about *leukemia* and *lymphoma*. Moreover, people often report the results of blood tests, a situation that increases the number of entities identified.

Terms tagged as *procedures* extracted from the corpus are mainly linked to oncologic diseases. About 2000 occurrences of *chemo* and *chemotherapy* were extracted. *Chemotherapy* is a significant procedure with numerous side effects. The fact that patients mention it at a high frequency shows that it is a treatment with a strong impact on quality of life and raises a lot of questions and worries for the patients involved. Social intervention procedures such as *listening* and *advice* are also frequent (see Table 3). This observation indicates that apart from technical information about treatment and surgeries, people also speak about the support they got during their disease or search for it in the community.

In medicine, symptoms can be difficult to differentiate from findings. This difference often resides in the context of the phenomenon. In the corpus, entities belonging to those categories as well as the side-effects category can be analyzed together to gain a better understanding of the results. Most frequent entities from these categories are closely related to the patient experiences and feelings. Concepts related to the feeling of fear are the most frequently present in this merged category: 7 out of 30 entities express feelings of fear or related with fear using the words *anxiety*, *stress*, *confused*, *scared*, *terrified*, *fear*, and *worry*. This is coherent with studies on cancer survivors that state the fear of cancer recurrence as almost universal among this population [48]. It appears that people with chronic conditions use social media to share feelings they have experienced. The chronic diseases selected in this corpus frequently imply severe impact on lifestyle and decrease life expectancy. Therefore, it is logical that *fear* and *anxiety* are prominent entities in the corpus.

Health-related quality of life in chronically ill patients is a known field in medical research since numerous years. Questionnaires such as the European Organisation for Research and Treatment of Cancer Quality of Life-C15-Palliative [49] or, more recently, the Functional Assessment of Cancer Therapy-General 7 [50] and Patient-Reported Outcomes Measurement Information System [51] are used routinely to assess it in those populations. When looking at the top concerns raised by patients suffering from cancer [50], it is interesting to note that they are in line with the top entities extracted from the corpus. More specifically, the most frequent nondisease entity extracted, *pain*, is a key item in multiple quality-of-life assessment questionnaires. This shows that the experiences that the patients share on social media platforms are coherent with what has been proven to have an impact on their life.

Overall, entities extracted from the corpus are coherent with similar studies conducted on health-related social media [27] and with validated evaluation of the quality of life of patients suffering from chronic diseases.

### Relation Extraction

The relation extraction performed on this corpus shows that the most highly represented relation type identified is the *disease-anatomy* relation (5550 occurrences). The pairs most frequently representing a disease and its localization are *cancer-lymph*, *asthma-lungs*, and *tumor-blood*. This suggests that people using social media platforms to speak about their chronic diseases are willing to explain which disease they suffer from as well as the location of the disease. This propensity is probably linked to the fact that such subreddits are used to share life experiences and to find people with similar backgrounds. This commonality can be reassuring and informative for a person suffering from the same disease. To find these people and knowledge, it is valuable to share the nature of the disease and its anatomical location.

The second most frequent entity pair is *disease-disease* (4668 occurrences). Pairs of entities such as *cancer-ALL* (acute lymphoblastic leukemia) and *asthma-allergy* are frequent. This co-occurrence of diseases might be related to the fact that chronic diseases often lead to complications and to other diseases. For example, *asthma-allergy* was perceived from the sentence “have allergy induced asthma.”

The third most frequent entity pair is *anatomy-anatomy* (3595 occurrences). When looking at specific occurrences of this pair, the pairs *neck-lymph*, *brain-lungs*, and *lungs-lymph* are frequent. Another entity pair, *head-hair* is related to people speaking about the side effects of chemotherapy.

Relations linking *diseases* to *procedures* are present at a high frequency in the corpus (2540 occurrences). When looking specifically in this category, it is clear that the most frequently identified disease in the corpus, *cancer*, is also most highly represented in those relations.

The relations extracted from the corpus demonstrate that patients with chronic diseases are willing to share detailed information about their health condition in a structured manner, describing thoroughly the disease, its location, the symptoms it caused, and the effect of treatment.

### Subject-Predicate-Object Entity Relation Extraction

The language patterns of subject-predicate-object relations demonstrate important characteristics of health social media language. As is apparent in the outputs, subject pronouns and object pronouns were frequently mentioned and were used mostly in the singular first-person pronoun, such as *I*, *me*, and *my*. These patterns are related to the way individuals share personal or family experiences (“I-had-a bad cold or sinus infection,” “Allergens-explains-my severe asthma,” “It-is making-my heartburn,” and “Anyone-develop-eczema”) and feelings (“I have a history of testicular cancer in family so Im pretty scared bht im hoping its nothing”). Also, patients or relatives, after having described their problem, treatment, and possible effects, often ask for advice, as shown in the following sentence:

I noticed the depression is occurring simultaneously with the increased asthma symptoms and was wondering if there is a correlation and if anyone else has experienced this?

### Social Media Language

Data derived from clinical narratives and research papers differ significantly from social media content. The language and style used by the authors as well as the content are different. From a linguistic point of view, medical blogs usually consist of syntactically correct sentences but can contain verbless clauses or sentences without subjects [52]. Abbreviations, enumerations, and citations of conversations, medical terms, and opinion-related words are used frequently in medical blog posts and websites. As stated in the study by Korkontzelos et al [53], “in social media, users rarely use technical terms.” Moreover, emoticons are very often used to convey emotion or to give contextual information to correctly understand a message (such as irony or sarcasm). The corpus processed in this research confirmed these observations.

### Limitations

There are 2 major limitations of the PKDE4J tool with regard to the objectives of this paper. First, PKDE4J was initially developed for the processing of well-structured biomedical texts and not for social media text. This issue has a relatively less impact on this paper given that the entity extraction task is based on dictionaries. However, for tasks such as part-of-speech and sentence parsing needed for the extraction of relations, the informality of social media text poses a challenge. Second, the lack of terms from the dictionaries as well as lexical and semantic ambiguities lowered the performance of the system. For instance, abbreviations and acronyms can have multiple interpretations, and this can lead to ambiguities. In the current version of the system, these types of ambiguities are not handled. Consequently, all occurrences of *ALL* found in the corpus were extracted, even those that do not refer to the disease *Acute Lymphocytic Leukemia*. Also, the lexical unit *back* has sometimes been falsely recognized as a body part.

### Conclusions

Data from social media platforms devoted to health can provide valuable information about the experiences of the patients involved. In this paper, we reported the application of an information extraction approach using the PKDE4J tool to detect, extract, and visualize chronic disease entities and relations and to identify characteristics of the social media language in a corpus collected from Reddit.

In the Results section, we showed which disease entities are frequently mentioned and which are the most frequent relation pairs. Relation extraction demonstrated that the most frequent relation pair is the *disease-anatomy* pair and the subject-object relation pattern in the social media language is the use of the first-person pronoun provided that people share personal experiences.

Although data privacy and information sharing is becoming a major concern in research and legal frameworks, such as the General Data Protection Regulation law, have begun to set boundaries for the storage and sharing of information generated by users, it is interesting that despite those concerns, users are willing to share private health information in open social networks.

Further research should focus on the enrichment of dictionaries and adaptation of rules to common usages of social media language and the processing of emoticons for the sentiment analysis task. Finally, the identification of the type of semantic relations and the evaluation on the relation extraction results should be performed to assess the performance of the system in this task.

## Abbreviations

ALL: acute lymphoblastic leukemia
AML: acute myeloid leukemia
BRCA2: breast cancer type 2
CLL: chronic lymphocytic leukemia
CML: chronic myeloid leukemia
COPD: chronic obstructive pulmonary disease
C-topic: conditional topic
DCIS: ductal carcinoma in situ
FWIW: For What it's Worth
GERD: gastroesophageal reflux disease
LDA: latent Dirichlet allocation
LOL: laughing out loud
MRSA: methicillin-resistant *Staphylococcus aureus*
TSH: thyroid stimulating hormone
